# P-1432. Communicating Readiness: Lessons Learned and Key Takeaways from an Ebola Vaccine Rollout at a Regional Emerging Special Pathogens Treatment Center

**DOI:** 10.1093/ofid/ofaf695.1619

**Published:** 2026-01-11

**Authors:** Arianna Boshara, Jacob Wiersch, Maria G Frank

**Affiliations:** Denver Health and Hospital Authority, Denver, CO; Denver Health and Hospital Authority, Denver, CO; Denver Health Hospital Authority, Denver, Colorado

## Abstract

**Background:**

The recombinant vesicular stomatitis virus-Zaire Ebola virus (rVSV-ZEBOV) vaccine is currently the only FDA-approved option for prevention of Ebola virus disease (Orthoebolavirus zairense) in individuals 12 months and older. As a replication-competent recombinant vector vaccine, it plays a key role in outbreak preparedness and healthcare worker (HCW) protection. In 2024, our Regional Emerging Special Pathogens Treatment Center (RESPTC) conducted a focused rollout of rVSV-ZEBOV to assess operational readiness and identify barriers to uptake among staff.

**Methods:**

Eligible HCWs were offered a single-dose vaccine through a coordinated process involving infection prevention, pharmacy, outpatient clinical operations, and the High-Risk Infection Team (HITeam). Observational feedback was collected through team debriefs and informal reports, and categorized across five key domains: engagement, logistics, pharmacy coordination, post-vaccine follow-up, and internal/external communications.

**Results:**

Of 30 interested HITeam members, only nine were vaccinated-underscoring gaps in communication methods. While clinic and pharmacy operations were well-coordinated, early scheduling was complicated by vaccine thaw times (30-45 minutes) and strict usage windows. Public-facing communication efforts were challenged by misinformation, including internal channels inaccurately describing the vaccine as “live,” which contributed to hesitancy. Despite limited recipient feedback, several noted the benefit of taking the following day off. The rollout also enhanced visibility of the HITeam and opened conversations across clinical departments.

**Conclusion:**

This pilot vaccination effort surfaced important lessons in operational preparedness and the critical role of tailored communication. In particular, emergency department (ED) staff-who are among the most likely to encounter undifferentiated febrile patients with potential VHF-must be prioritized in future outreach. Embedding vaccine education into ED workflows and ensuring clarity around risk, safety, and logistics will be essential to strengthening trust and improving uptake in future preparedness efforts.

**Disclosures:**

All Authors: No reported disclosures

